# Application of Simulated Arms with Real-Time Pressure Monitor in Casting and Splinting by Physiological Sensors

**DOI:** 10.3390/s21175681

**Published:** 2021-08-24

**Authors:** Hsuan-Kai Kao, Yi-Chao Wu, Chi-Heng Lu, Zhong Hua, Mei-Chuan Chen, Chiu-Ching Tuan

**Affiliations:** 1 Department of Orthopedic Surgery, Chang Gung Memorial Hospital at Linkou, Taoyuan 33305, Taiwan; samiyadon@cgmh.org.tw (H.-K.K.); cat0520307@cgmh.org.tw (M.-C.C.); 2 Bone and Joint Research Center, Chang Gung Memorial Hospital, Taoyuan 33305, Taiwan; 3 College of Medicine, Chang Gung University, Taoyuan 33305, Taiwan; 4 Interdisciplinary Program of Green and Information Technology, National Taitung University, Taitung 95092, Taiwan; alanwu@nttu.edu.tw; 5 Department of Electronic Engineering, National Taipei University of Technology, Taipei 10608, Taiwan; chiheng@cgmh.org.tw (C.-H.L.); t105368064@ntut.edu.tw (Z.H.); 6 Department of Radiation Oncology, Chang Gung Memorial Hospital at Linkou, Taoyuan 33305, Taiwan

**Keywords:** physiological sensors, cast fixation, fractures, simulated arm, real-time, casting and splinting, skin surface, radial styloid, ulnar styloid, swathed cast

## Abstract

In the real condition, the small sensor found it difficult to detect the position of the pressure sore because of casting displacement clinically. The large sensor will detect the incorrect pressure value due to wrinkles without close to arm. Hence, we developed a simulated arm with physiological sensors combined with an APP and a cloud storage system to detect skin pressure in real time when applying a short arm cast or splint. The participants can apply a short arm cast or splint on the simulative arm and the pressure in the cast or splint could be immediately displaced on the mobile application. The difference of pressure values from six pressure detection points of the simulated arm between the intern and the attending physician with 20-year working experience were 22.8%, −7.3%, 25.0%, 8.6%, 38.2%, 49.6%, respectively. It showed that the difference of pressure values in two farthest points, such as radius stab and ulnar styloid, was maximal. The pressures on the skin surface of the short arm cast were within acceptable range. Doctors would obtain reliable reference data and instantly understand the tightness of the swathed cast which would enable them to adjust it at any time to avoid complications.

## 1. Introduction

Combining medical things with the IoT is a trend recently and also the object of this paper. Hence, an application of simulated arms with real-time pressure monitor in casting and splinting by physiological sensors was proposed in this paper. This system was included medical, physiological sensors, cloud database, network, and mobile device.

Fractures are common injuries, and cast or splint immobilization remains the primary treatment. Casting is not without risks and complications. The risk of morbidity is higher when casts are applied by less experienced practitioners [1]. The poor blood circulation, compartment syndrome, and pain will occur when the cast is too tight. Moreover, the patients may need amputation in serial condition [2]. However, the fixed therapeutic effect could not be achieved due to a loose cast. Hence, the efficient immobilization is important for the fractures in patients.

The behavior of medical health care was radically changed by combining the sensors with everyday general purpose items, such as shoes and clothes. In [3], the reel-to-reel fabrication of the strain sensor was integrated with smart insole for gait monitoring. In [4], the wearable device with silver-nanowire-coated wool fibers was designed to detect the breathing and finger movement. In [5], the forward-sensing fiber-optic pressure sensor was used for examining the intramuscular pressure. In [6], the TekScan FlexiForce A201 sensor was used to measure the load force in different points of plantar surface for assessing the load force distribution of feet and ankles with different kinds of plaster. Due to the potential applications in both medical health care and human-computer interaction, the flexible pressure sensor was taken attention recently. However, it still could be used widely since the manufacturing process was complex and the hardware cost was high [7,8]. Hence, the resistive pressure sensor called RP-L TDS was used in the experiment first.

The thickness of RP-L TDS was 0.4 mm. The detection sensitivity of RP-L TDS was higher and the detection range of RP-L TDS was wider. The value of pressure detected by RP-L TDS was from 10 to 20 g. In RP-L TDS, the rectangular area all could be used for pressure sensing. However, it only could detect the sum of all sensing points. It cannot detect the individual value of each sensing point. Hence, RP-L TDS was waived to be used. Then, we used the thin film pressure sensor called Flexiforce A301 in the experiment. The length of Flexiforce A301 was 25.4 mm prone to be wired. The detection sensitivity of Flexiforce A301 was higher. The value of pressure detected by Flexiforce A301 was from 0 to 454 g. However, the sensing area of Flexiforce A301 was too small to not be triggered, such as 0.7 cm^2^. Hence, Flexiforce A301 was waived to be used. Since the detection sensitivity of Flexiforce A401 was also higher and the sensing area was larger than the sensing area of Flexiforce A301 to be triggered, around 5 cm^2^, Flexiforce A401 was used in our paper finally.

When teaching cast skills, the young physicians are generally guided by the subjective experience without the scientific data to calculate the tightness of cast. Young physicians usually find it difficult to know how tight is optimal when applying a cotton and plaster cast. Young physician competence to perform a cast or splint is often presumed and not confirmed by objective measures [9].

The purpose of this study was to develop a simulated arm with pressure sensors which can detect real-time skin pressure when applying a short arm cast or splint. In addition, we also bring the simulated arm to casting and splint workshops to see how it works. Fractures are common injuries, and cast or splint immobilization remains the primary treatment. Casting is not without risks and complications. The risk of morbidity is higher when casts are applied by less experienced practitioners [1].

In this paper, we aimed to develop a simulated arm with pressure sensors which can real-time detect skin pressure when applying a short arm cast or splint. In addition, we also bring the simulated arm to casting and splint workshops to see how it works.

In the real condition, it is difficult to detect the position of pressure sore clinically due to the casting displacement, since the size of the sensor is too small. However, it is hard to get close to the arm when the size of the sensor is large. A wrinkle is often created by the large sensor which detects the incorrect pressure value. Hence, this paper aimed to address the above issues to improve the existing system to help the interns overcome the casting training problems caused by sensing from sensors. In addition, the function and UI of APP were improved, and a cloud storage system was added in this paper. Hence, the interns could obtain the pressure value by sensors on casting in real time and could query the record of casting pressure value to improve the casting next time.

## 2. Materials and Methods

**Figure 1:**
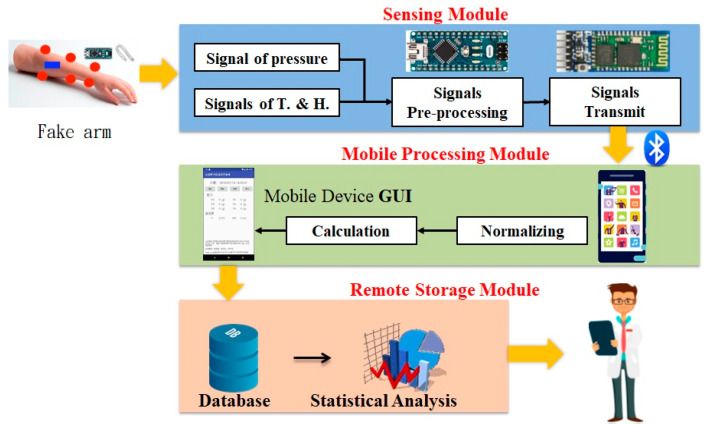
System architecture.

In this paper, a system for measuring the humidity, temperature, and pressure caused by the cast on the skin was proposed to provide an objective factor of casting on a practice simulated arm for young physicians in real-time. The data detected by the system will be uploaded to the database to observe the process difference between the clinical practice and teaching. In this system, the physiological sensors’ deployment, sensing module, and embedded broad for controlling and communication were required firstly. Secondly, the readable pressure data were obtained by embedded broad with the processing module. Then, these data were transmitted to the smart mobile device through Bluetooth. Finally, the data, such as humidity, pressure, and temperature, were uploaded to the database via the Internet in real time. To be observed easily, the different kinds of data are displayed by different colors. The humidity and temperature sensors were deployed close to the affected area. The pressure sensor was deployed in an area where pressure is suspected. The system architecture we proposed in this paper was shown in Figure 1.

**Figure 2:**
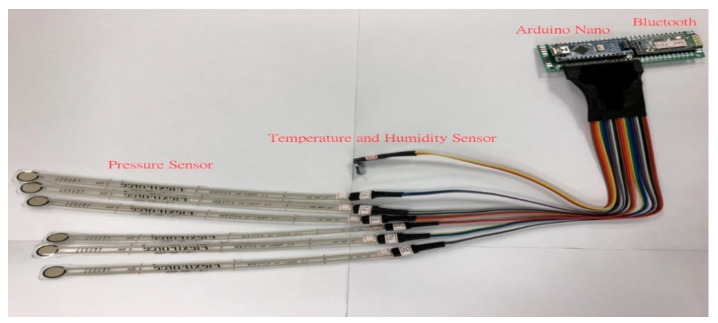
Sensing module.

The differences of signals of the humidity, pressure, and temperature by the external environment were converted by the sensing module. Then, these converted signals are transmitted to the mobile device via Bluetooth. The sensing module was shown in Figure 2.

**Figure 3:**
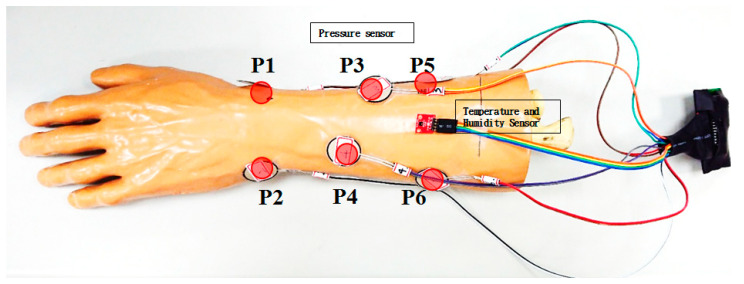
Location map of pressure, temperature, and humidity detection points of simulated arm.

Total eight detection points were deployed on the radial and ulnar side of the simulated arm (Sawbones, Vashon Island, WA, USA). Six of them were the pressure sensors, as listed at Table 1. Two of them were the temperature and humidity sensors, respectively, as shown in Figure 3. The points of pressure sensors were indicated as P1–P6.

The 6 pressure sensors are placed on the most prominent bone points of the simulated arm. The most distal 2 pressure sensors are placed over the radial styloid and ulnar styloid. These 2 points are the most bone prominence and are the most common sites of pressure sores clinically. The other 4 pressure sensors are placed along the radius and ulna. The skin under these 4 points is thin and easily injured in the cast. Therefore, these 6 locations are the most dangerous points for developing pressure sores clinically. We use the pressures of these 6 points to represent the general condition in the cast.

When a pressure sore occurs, the skin is injured or infected. Signs and symptoms of pressure sores include fever, pain, redness, swelling, warmth of the area, and serosanguineous/purulent discharge. Hence, we add the temperature/humidity to be able to detect serious skin damage in the cast early. In [9], the feasibility verification and performance evaluation of temperature/humidity sensing were examined in the experiment. The points of temperature and humidity sensors were deployed at the halfway point of the mid-line of the simulated arm. Since the experiment was processed in a simulated arm, no volunteer was needed. The time of measuring was varied based on the individual cast swath time and the 10 Hz sampling frequency. We have completed pressure, temperature, and humidity verification in the previous study.

However, the experimental results showed that the temperature/humidity sensing addressed nothing for the pressure to the skin. Moreover, this paper focused on the training of interns, APP improvement, and cloud database design. The temperature/humidity sensing was thus not considered in this paper. Therefore, we only measured the pressures when participants were applying a cast. The participants cannot see the pressure measured by the sensors when they are applying a cast. The APP designed in this paper could show the pressure of six points in the simulated arm. The sensing data were transmitted to the cloud database via the Internet. Hence, the interns could carry out the plaster coating without any tool. The interns also could obtain the status of force from the scientific data by APP in real time.

In this paper, we proposed an application of simulated arms with a real-time pressure monitor in casting and splinting by physiological sensors based on [10]. In [10], the humidity, pressure, and temperature could be detected in the simulated arm. However, the better or optimal locations for the physiological sensors to detect the humidity, pressure, and temperature on the simulated arm were not simulated in [10]. In this paper, we found that the locations of the physiological sensors will affect the accuracy rate of the experimental results. Hence, we brought the simulated arms to the casting and splinting workshops in our hospital to collect more data to determine the best locations for the physiological sensors. Moreover, the UI of APP was also improved to be observed by the patients and medical staff in APP in real-time through the Internet in this paper.

In [10], the value of pressure was from 0 to 35 g in general condition. The value of pressure was more than 35 g and may be up to 350 in abnormal stress. To ensure the reliability of our pressure system, the verified experimental range was up to 500 g with one weight with 10 g, two weights with 20 g, one weight with 50 g, two weights with 100 g, and one weight with 200 g, respectively. Firstly, the first segment transferred function where the pressure value measured from 0 to 50 g was derived. The second segment transfer function with a pressure value measured from 50 to 150 g was derived. The third segment transfer function with a pressure value measured from 150 to 300 g was derived. Finally, the fourth segment transferred function with the value of pressure measured from 300 to 500 g was derived. Each segment transferred function was measured at a 10 Hz sampling frequency for 10 s. Hence, one segment transfer function had 100 records in a round. Since each segment transfer function was calibrated with six rounds, one segment transfer function had a total of 600 records. By calculating the deviation between the value of each segment transfer function and the value of the weight, the minimal deviation was 1.31% and the maximal deviation was 1.82%. The deviation of each segment transfer function was within 2%. Hence, the pressure sensors used in this paper could be calibrated and verified. The pressure was detected by strain effect in our pressure sensing module. In the strain effect, the voltage was varied by the deformation of strain gauges due to the impact of the component. The stable DC voltage of the sensor was supplied by a rechargeable battery. The output voltage signal was varied by the varied impedance of the pressure sensing circuit. The voltage signal was then transferred into the pressure signal. The specifications of the pressure sensor showed that the curve transferred from voltage into pressure was non-linear. It will have the deviation while transferring voltage into pressure by transferred function with a single conversion coefficient. Hence, the transferred function with four segments conversion coefficient was proposed to transfer voltage into pressure. To avoid selecting an unsuitable segment transferred function to generate the deviation, it will select the suitable segment transferred function to transfer voltage into pressure by analog-to-digital converter in our pressure sensing module automatically.

**Figure 4:**
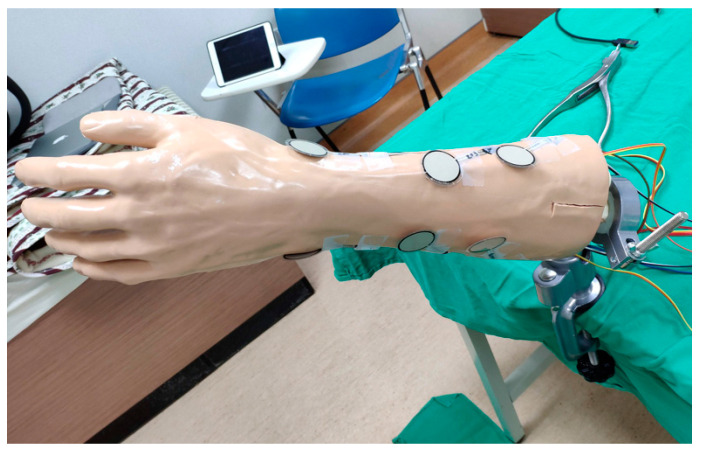
The simulated arm was fixed to a clamp which was mounted to a bench top.

We asked the hospital whether this experiment required the Institutional Review Board (IRB) or not before the experiment was executed. The answer from the hospital was that the IRB was not required, since we brought the simulated arms to the casting and splinting workshops in our hospital. The simulated arm was fixed to a clamp which was mounted to a bench top, as shown in Figure 4. The participants can apply a short arm cast or splint on the simulated arm and the pressure in the cast or splint can immediately be displayed on the mobile application.

While the FlexiForce A401 (Tekscan Inc., South Boston, MA, USA) sensor was used in the simulated arm, it was fixed by using 3M breathable tape. Hence, the redundant value was generated by bending or stress as mentioned in the reviewer’s comment. Hence, this redundant value will be neglected and the initial value of the sensor will be returned to zero by the APP to solve the above problem.

**Figure 5:**
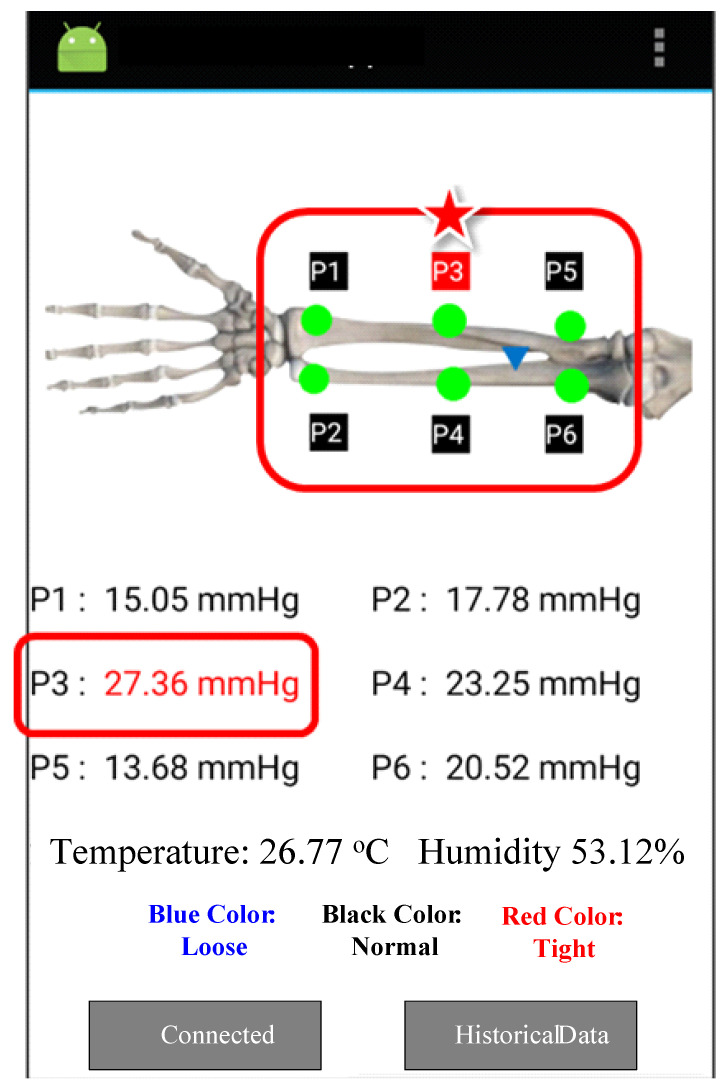
The screenshot of the mobile application when performing a short arm cast.

We set the optimal pressure under the cast to 8–25 mmHg, according to previous studies [11,12,13]. When the pressure is within 8–25 mmHg, the color of the pressure number on the mobile application is black. When the pressure is less than 8 mmHg, the cast is classified as too loose. The color of the pressure number on the mobile application will become blue. When the pressure is greater than 25 mmHg, the cast is classified too tight. The color of the pressure number on the mobile application will become red on the mobile application, as shown in Figure 5.

**Figure 6:**
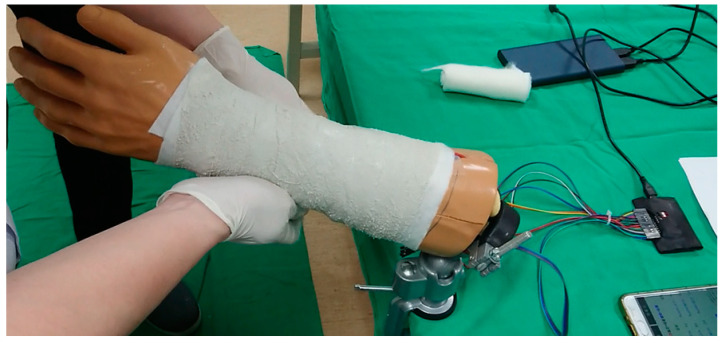
The participant applies a short arm plaster cast to the simulated arm.

The participants of the casting and splinting workshop are sixth-year medical students. Before applying a cast or splint, they received oral instruction and demonstration of the cast and splint application by senior orthopedic physicians. Then, they were asked to apply a short arm plaster cast on the simulated arm, as shown in Figure 6. The pressures of the 6 detection points were recorded continuously. After applying the short arm plaster cast, the participants were asked to fill the questionnaire about their satisfaction with this system.

## 3. Results

**Figure 7:**
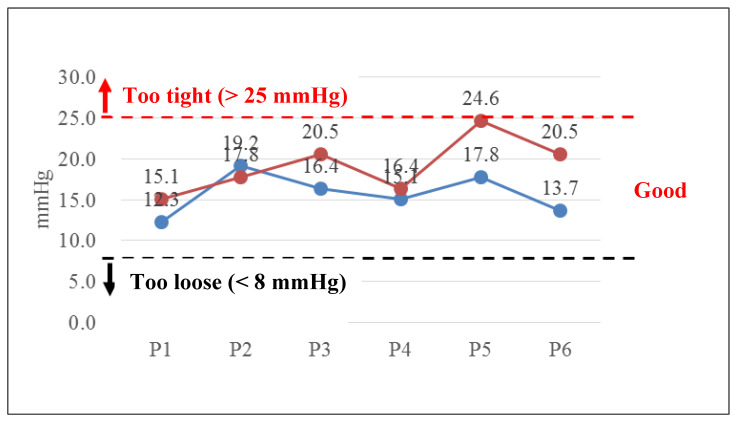
The pressures of P1-P6 when attending physicians and resident applying a short arm cast.

The casting and splinting application workshop was held in June 2019. The instructors were one orthopedic attending physician and one third-year orthopedic resident. Twenty-three 6th-year medical residents attended the workshop and 13 of them used the simulated arm to evaluate the pressures while performing a short arm cast. The pressures on the skin surface when attending physician and resident performing a short arm cast were recorded as baseline, as shown in Figure 7. All pressures detected during performing a short arm cast were within acceptable range.

The difference of the pressure values measured from P1 to P6 of the simulated arm between the intent and the attending physician with 20-year working experience were 22.8%, −7.3%, 25.0%, 8.6%, 38.2%, 49.6%, respectively, in the experiment. It showed that the difference of the pressure values in two farthest points, such as radius stab and ulnar styloid, was maximal. These two points were the most protruded bones. It was also the location of a pressure sore clinically.

**Figure 8:**
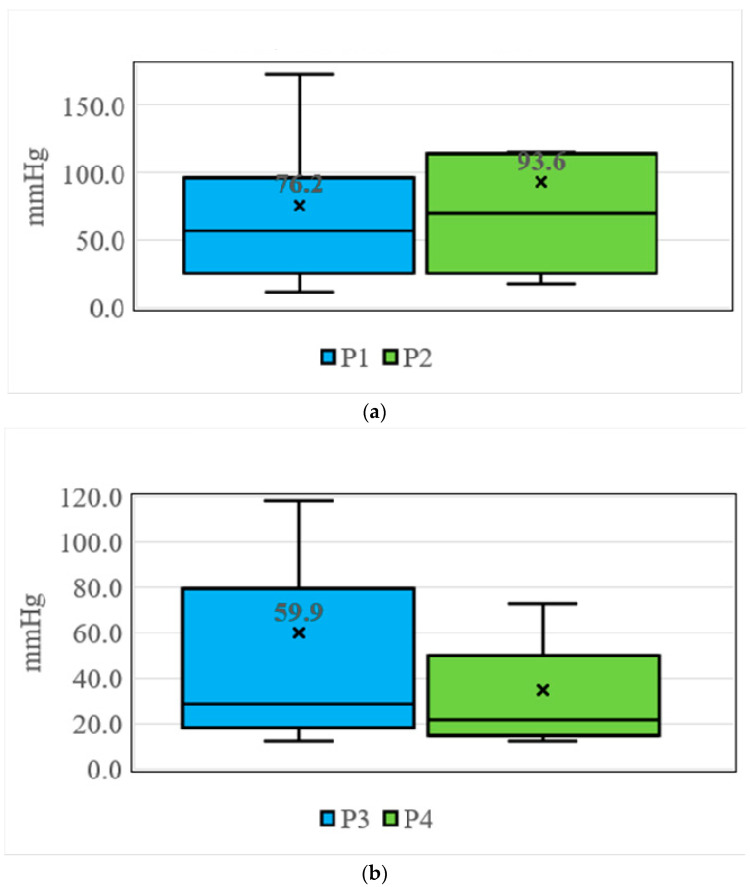

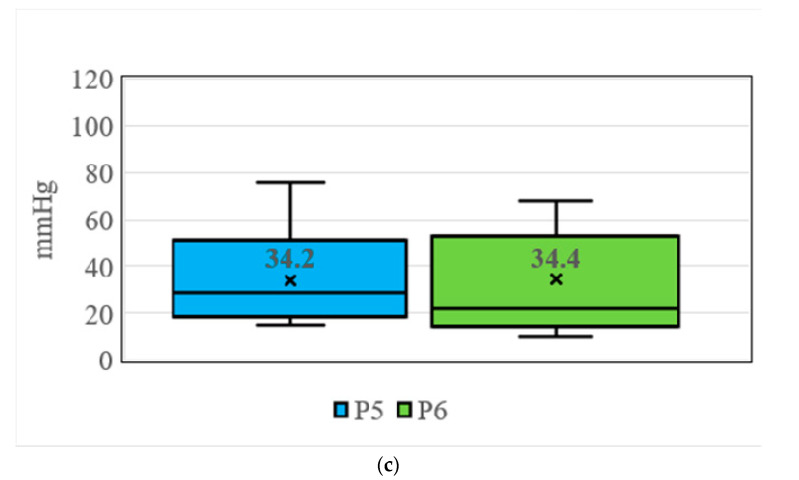
(**a**) Pressures detection over the P1 and P2 sensors. (**b**). Pressures detection over the P3 and P4 sensors. (**c**). Pressures detection over the P5 and P6 sensors.

The participants in Figure 7 are attending physicians and residents. They are all experienced orthopedic doctors. However, the participants in Figure 8 are sixth-year medical students. They are not orthopedic doctors and not familiar with how to apply a cast. Therefore, they applied too much pressure when performing a short arm cast.

On the P1 and P2 positions, the mean pressures were 76.2 mmHg and 93.6 mmHg when the sixth-year medical students applied the short arm plaster cast. The pressures detected were higher than safe range. The highest pressure detected was from P2 sensor which is located over the ulnar styloid, as shown in Figure 8a. On the P3 and P4 positions, the mean pressures were 59.9 mmHg and 34.6 mmHg when the sixth-year medical students applied the short arm plaster cast. The pressures were lower than P1 and P2, but still higher than the safe range, as shown in Figure 8b. On the P5 and P6 positions, the mean pressures were 34.2 mmHg and 34.4 mmHg when the 6th-medical students applied the short arm plaster cast. The pressures were lower than P3 and P4, but still higher than safe range, as shown in Figure 8c.

## 4. Discussion

Current training programs for cast or splint application largely depend on senior orthopedic doctors’ enthusiasm. Younger doctors learn and practice cast or splint application on real patients under the supervision of senior doctors. However, due to reduced work hours and the concern for patient safety, this training method becomes more challenging. Therefore, simulation for learning technical skills has occurred [14].

Distal radial fracture is a common fracture pattern, which accounts for 20% to 36% of all fractures. The distal radial fractures are usually treated by closed reduction and cast or splint immobilization [15]. Approximately one-third of all distal radial fractures re-displace [16]. Risk factors for re-displacement include inadequate reduction, poor cast molding, and the inexperience of junior trainees [11,12]. Therefore, cast application skills are fundamental to orthopedic practice.

When teaching cast skills, one of the most challenging issues is the pressure which physicians apply the cotton and plaster cast. Senior doctors usually demonstrate cast techniques by their experience. They usually warn young doctors not to tighten when rolling the cotton and plaster cast. However, this method is subjective and lacks a precise pressure number. Young doctors usually find it difficult to know how tight is optimal when applying a cotton and plaster cast. Therefore, we developed a simulated arm with 6 pressure sensors which can detect real-time pressure when applying a short arm cast or splint [10]. We utilized this simulated arm in cast and splint workshops for 6th-year medical students.

The teachers of the workshops were an attending orthopedic physician and an orthopedic resident. They were asked to apply a short arm cast before the workshops. All pressures detected during application of the short arm cast were within an acceptable range. This means that the senior physicians have qualified cast skills, and the pressure detection function of the simulated arm is adequate.

The mean pressures when the 6th-year medical students applied the short arm cast were higher than senior physicians. Even though the senior physicians demonstrated how to adequately roll the cotton and the plaster cast, the students still apply too much pressure when performing a short arm cast. The higher pressure under the cast can injure the soft tissue in the cast. This simulated arm can help senior physicians teach adequate pressures when applying a cast. The simulators can offer effective training by affording limitless practice opportunities in a low-risk environment [17,18,19].

The two highest pressures detected were located on the radial styloid and ulnar styloid. In these two areas, the radius and ulna are prominent and soft tissues are vulnerable. Young doctors must be warned that these two areas are most susceptible to physical pressure. They must apply enough cotton over these areas and not to apply too much pressure on them.

The incidence of cast-related complications was 5.6 to 13.6 per 1000 casts applied [19,20]. The most common location of the skin complication was on the heel [20]. Julie Balch Samora et al. developed a quality improvement model to reduce cast complications. This quality improvement model includes Plan-Do-Study-Act cycles. With the quality improvement intervention, the cast complication rate was reduced from 5.6 to 1.61 complications per 1000 applications [20]. Our training program with the simulated arm is also a quality improvement method. With the real-time pressure detection sensors, the learners can understand how much pressure is adequate when applying a short arm cast. Therefore, the pressure on the skin is reduced and the complication rate can also be reduced.

## 5. Conclusions

Combining IoT and intelligent medical is the main object in this paper. Cast fixation is a specialized clinical skill commonly used for the treatment of fractures. However, it could cause many complications due to careless handling. At present, when doctors help patients to swathe a cast, they could only decide how tight the cast should be based on their own experiences without any objective and reliable equipment to inform them of the pressure within it, which often results in patient injuries from a too tight or too loose cast. Moreover, the existing studies addressed nothing concerning the pressure sensing of casting and the interns’ training on casting, jointly. The final object of this paper aimed to reduce the pressure sore in fracture patients. However, the flexible pressure sensor still could not be used due to the immature manufacturing technology and high hardware cost.

Therefore, we have developed a novel simulated arm with physiological sensors, such as six pressure sensors, an APP, and a cloud storage system in this paper. The simulated arm can help senior physicians teaching cast skills, especially with the pressure when applying the cast. In addition, the radial styloid and ulnar styloid are two vulnerable areas which have higher pressure when applying a short arm cast. Orthopedic physicians must be aware of the problem and be cautious when applying the cast. The interns could improve their casting ability by querying the record in APP with cloud storage system next round after the experiment result of this round was completed.

A simulated arm with physiological sensors, such as pressure sensors, was proposed to detect skin pressure in this paper. It was also applied for the simulated arm in casting and splinting workshops to know if this device could help senior physicians teaching cast skills. We applied the simulated arm to the splinting and casting workshops. We fixed the sensors to the simulated arm. The purposes of the sensors are real-time monitoring pressure in the cast. Therefore, the sensors will be kept in the cast. However, the materials of the sensors are not biocompatible. It cannot be put on the skin too long. If we use the sensors in real patients, we will put the sensors on the cotton roll which is used to cover and protect the skin. Moreover, it could train the interns to examine the status of force while these interns carried out the plaster coating of the simulated arm by APP in each round.

In the experiment, the instructors of the casting and splinting workshops are one orthopedic attending physician and one third-year orthopedic resident. The participants of the casting and splinting workshop are sixth-year medical students. Since the experiments were processed on the simulated arm, no biocompatible problem existed in this paper. Hence, the IRB was not required.

In the future, capturing the pressure value of casting on the real arm by applying to IRB will be the first object. Secondly, we will detect the pressure value of casting on the simulated arm in more positions. Finally, we will use other types of sensors to capture more accurate pressure values of casting.

## Figures and Tables

**Table 1:** Locations of 6 pressure sensors.

Sensor Code	Location
P1	Radial styloid
P2	Ulnar styloid
P3	Radial shaft
P4	Ulnar shaft
P5	Proximal radius
P6	Proximal ulna

## Data Availability

Not applicable.
